# Prospective Associations Between Working Time Arrangements and Psychiatric Treatment in Denmark: Protocol for a Cohort Study

**DOI:** 10.2196/18236

**Published:** 2020-06-15

**Authors:** Harald Hannerz, Karen Albertsen, Martin Lindhardt Nielsen, Anne Helene Garde

**Affiliations:** 1 The National Research Center for the Working Environment Copenhagen Denmark; 2 TeamArbejdsliv Valby Denmark; 3 Lægekonsulenten dk Viby J Denmark

**Keywords:** occupational health, long working hours, night shift work, mood disorders, anxiety, stress-related disorders, psychiatric hospital treatment, prescription drugs, psychotropic medicine

## Abstract

**Background:**

The burden of mental ill health in working-age populations has prompted research on possible links between work-related factors and mental ill health. Long working hours and night shift work are some of the factors that have been studied in relation to the risk of developing mental ill health. Yet, previous studies have not generated conclusive evidence, and further studies of high quality are needed.

**Objective:**

This study aims to investigate the prospective association between working time arrangements and mental health in terms of psychotropic drug usage or psychiatric hospital treatment in the general working population of Denmark.

**Methods:**

Data on total weekly working hours in any job and night shift work from the Danish Labor Force Survey 2000–2013 will be linked to data from the Psychiatric Central Research Register (expected 2400 cases during 700,000 person years at risk) and National Prescription Registry (expected 17,400 cases during 600,000 person years at risk). Participants will be followed for up to 5 years. We will use Poisson regression to separately analyze incidence rates of redeemed prescriptions for psychotropic medicine and incidence rates of psychiatric hospital treatment due to mood disorders, anxiety disorders, or stress-related disorders as a function of weekly working hours and night shift work. The analyses will be controlled for sex, age, calendar time of the interview, and socioeconomic status.

**Results:**

This is a study protocol. Power calculations indicate that the study has sufficient statistical power to detect relatively small differences in risks and minor interactions (eg, ~90% power to detect a rate ratio of 1.1 for psychoactive medication use). We expect the analyses to be completed by the end of 2020 and the results to be published in 2021.

**Conclusions:**

In this study protocol, all hypotheses and statistical models of the project have been completely defined before we link the exposure data to the outcome data. The results of the project will indicate to what extent and in what direction the national burden of mental ill health in Denmark has been influenced by long working hours and night shift work.

**International Registered Report Identifier (IRRID):**

DERR1-10.2196/18236

## Introduction

### Background

The average prevalence of mental ill health in the working-age population of Organization for Economic Co-operation and Development (OECD) countries has been estimated at 20% [1]. It has, moreover, been estimated that approximately 30% to 50% of all new disability benefit claims in OECD countries can be attributed to mental ill health [1]. The massive burden of mental ill health in working-age populations has prompted research on possible links between work-related factors and mental ill health [2,3]. Long working hours and shift or night work are some of the factors that have been studied in relation to the risk of developing mental ill health.

A theoretic argument for an adverse effect of long working hours on mental health is their association with short sleep [4-6] and fatigue due to insufficient recovery between work shifts [4,6-9], which are known risk factors for mental ill health [10-16]. The same argument may be applied to shift work, which, especially if it includes night shifts, disrupts the circadian chronobiologic rhythm and increases the risk of sleeping problems and fatigue [17-19]. Prolonged working hours as well as shift work may, however, generate extra income compared with equivalent daytime work without overtime, and a higher income has been associated with a decreased risk of developing psychological distress [20], depressive symptoms [21], and depression [22]. There are, in other words, theoretical arguments for beneficiary as well as detrimental mental health effects of long working hours and shift work.

Most of the published prospective studies on the relationships between long working hours and mental ill health have, however, been underpowered to such a degree that they do not impart any meaningful information [23]. The few studies in which the statistical power has been acceptable have reported rate ratios (RRs; for long vs normal working hours) that are close to unity in study populations from Europe, North America, and Australia and slightly elevated in study populations from Asia [3,24]. The meta-analysis by Virtanen et al [24] included 28 cohort studies with a total of 189,729 participants from 35 countries and estimated the RR for development of depressive symptoms between workers with long vs standard working hours at 1.50 (95% CI 1.13-2.01) in Asia, 1.11 (95% CI 1.00-1.22) in Europe, 0.97 (95% CI 0.70-1.34) in North America, and 0.95 (95% CI 0.70-1.29) in Australia. Recent literature reviews on shift work and mental health [3,18,25-27] do not yield any clear evidence of a prospective association between shift or night work and mental disorders.

### Aims and Objectives

In a previous study of prospective associations between long working hours or shift work and redeemed prescriptions for psychotropic medicine among employees in the general population of Denmark [23,28,29], we did not find any statistically significant effects after adjustment for multiple comparisons. However, our previous study could not reject the possibility that excessive overtime work (>48 vs 32-40 working hours a week) is associated with a clinically important effect (RR 1.15, 95% CI 1.02-1.30). Thus, although not statistically significant, our primary analyses suggested that the average risk among employees with excessive overtime work might be slightly higher than it is among employees with normal working hours. Our secondary (hypothesis-generating) analyses suggested that excessive overtime work may be an important risk factor among shift workers (RR 1.51, 95% CI 1.15-1.98) [23]. Our secondary analyses suggested, moreover, that the RR among shift vs non-shift workers was markedly higher for redeemed prescriptions of antidepressants (RR 1.23, 95% CI 1.08-1.40) than it was for redeemed prescriptions of anxiolytics (RR 0.86, 95% CI 0.72-1.02) [29].

In the present project, we will test/retest some of the hypotheses that were generated or tested in our previous study, in a data set that is independent of and larger than those previously used. The target population (employees in the general population of Denmark) is the same, and the working hours of the participants will be categorized in the same way as in our previous study (32-40 hours/week, 41-48 hours/week, and 49-100 hours/week). We will, however, not be able to reproduce the shift work categories, which in our previous study were defined as “fixed night shifts or rotational shift work schedules” vs “fixed day, morning, or evening shifts.” Instead, we will look at the contrast “schedules that include night shift work” vs “other work schedules (including non-night shift work and evening work)”.

## Methods

### Ethics Approval

The study will comply with The Act on Processing of Personal Data, Denmark (Act No. 429 of May 31, 2000), which implements the European Union Directive 95/46/EC on the protection of individuals. The data usage is approved by the Danish Data Protection Agency, file number 2001-54-0180. The ethical aspect of the project was approved by Statistics Denmark, account number 704291.

### Clinical Endpoints

The following endpoints will be regarded: redeemed prescriptions for any type of psychotropic medicine (ie, drugs in the ATC-code category N05 [psycholeptica] or N06 [psychoanaleptica]); redeemed prescriptions for antidepressants (ATC-code: N06A); redeemed prescriptions for anxiolytics (ATC-code: N05B); redeemed prescriptions for hypnotics and sedatives (ATC-code: N05C); psychiatric hospital treatment with a mood disorder, anxiety disorder, or stress-related disorder (ICD-10: F30-F41 or F43) as the principal diagnosis; psychiatric hospital treatment with a mood disorder (ICD-10: F30-F39) as the principal diagnosis; and psychiatric hospital treatment with an anxiety-related or stress-related disorder (ICD-10: F40, F41, or F43) as the principal diagnosis.

The following mental disorders are included in the case definitions: F30 Manic episode, F31 Bipolar affective disorder, F32 Depressive episode, F33 Recurrent depressive disorder, F34 Persistent mood [affective] disorders, F38 Other mood [affective] disorders , F39 Unspecified mood [affective] disorder, F40 Phobic anxiety disorders, F41 Other anxiety disorders, and F43 Reaction to severe stress, and adjustment disorders.

### Hypotheses Tests

In this section, we list the statistical significance tests of the study. All of the tests will be adjusted for age, sex, socioeconomic status (SES), and calendar year of interview. Moreover, the tests for effects of weekly working hours will be adjusted for night shift work, and the tests for effects of night shift work will be adjusted for weekly working hours.

#### Incident Use of Psychotropic Medicine

With regard to prospective associations between long working hours or night shift work and redeemed prescriptions for any type of psychotropic medicine, we will test the following effects for statistical significance at an α of .01: main effect of weekly working hours, effect of interaction between age and weekly working hours, effect of interaction between sex and weekly working hours, effect of interaction between SES and weekly working hours, effect of interaction between night shift work and weekly working hours, main effect of night shift work, effect of interaction between age and night shift work, effect of interaction between sex and night shift work, and effect of interaction between SES and night shift work.

The familywise error rate denotes the probability of at least one false positive result among a family of related hypothesis tests, under the overall null hypothesis of no association. Since each of the hypotheses are tested at the significance level of .01 and 5 hypotheses are tested for each of the factors “long working hours” and “night shift work,” the familywise error rates for effects on incident use of psychotropic medicine will be ≤0.05 for each of these factors.

#### Psychiatric Hospital Treatment

Hazard ratios for incident use of psychotropic medicine are often used in occupational health research as a proxy measure for hazard ratios of mental ill health [30]. A large proportion of people who use psychotropic medicine do so to cope with sleeping problems [31]. Sleeping problems may be consequences (symptoms) of mental health problems, such as depression, anxiety disorders, and stress-related disorders [32]. They may, however, also be caused by factors that are unrelated to mental health, which may be especially true among shift or night workers, where sleeping problems often occur as a natural consequence of a disrupted circadian rhythm [17]. Sleeping problems may be treated not only with hypnotics and sedatives but also with anxiolytics and antidepressants [31]. Increased rates of incident use of psychotropic medicine are therefore not necessarily the same as increased rates of mental ill health. We will therefore supplement our analyses of psychotropic medicine usage with an examination of hazard ratios for psychiatric hospital treatment, although the threshold for this type of treatment is much higher. Unfortunately, valid data for diagnoses and treatment of mental disorders by general practitioners or private psychiatrists or psychologists in Denmark are not available.

With regard to prospective associations between long working hours or night shift work and psychiatric hospital treatment due to a mood disorder, anxiety disorder, or stress-related disorders, we will test the following effects for statistical significance at an α of .05: main effect of weekly working hours and main effect of night shift work.

Psychiatric hospital treatment is a relatively rare event, which makes the statistical power too low to allow testing for interaction effects.

#### Odds of Antidepressants vs Anxiolytics

Antidepressants are primarily designed to treat depressive mood disorders but can also be used for the treatment of anxiety and sleeping disorders [31]. In our previous study of employees in Denmark [29], we did not find any significant effect of shift work on incident use of psychotropic drugs when all types of psychotropic drugs were combined into a single outcome. We observed, however, a difference between shift workers and non-shift workers in the distribution of prescriptions for antidepressants and anxiolytics. The odds that a prescription was for antidepressants rather than for anxiolytics were markedly higher among shift workers than they were among non-shift workers.

The observed difference may have been due to chance, different practices for prescription of psychotropic drugs to shift workers compared with non-shift workers to avoid side effects of anxiolytics that may impede wakefulness during night shifts, or an increased risk of mood disorders combined with a decreased risk of anxiety and stress-related disorders among shift workers compared with non-shift workers.

In the present study, we will try to shed some light on this issue.

Among the employees who redeem a prescription for either antidepressants or anxiolytics, we will test if the odds for antidepressants vs anxiolytics differ between employees with and without night shift work.

Among the employees who receive hospital treatment for a mood disorder, anxiety disorder, or stress-related disorder, we will test if the odds that the treatment concerns a mood disorder vs an anxiety disorder or stress-related disorder differ between employees with and without night shift work.

Both tests will be performed at the significance level .05.

If the odds for antidepressants vs anxiolytics are significantly higher among the night shift workers, then it is unlikely that our previous observation was due to chance (hypothesis A).

If the odds for antidepressants vs anxiolytics are significantly higher while the odds for mood disorders vs anxiety disorders and stress-related disorders are lower among the night shift workers than they are among the non-night shift workers, then we have generated support for the hypothesis of prescription bias (hypothesis B).

If the odds for mood disorders vs anxiety disorders and stress-related disorders are significantly higher among the night shift workers than they are among the non-night shift workers, then we have generated support for hypothesis C.

### Data Material

Our project will be based on interview data from the Danish Labor Force Survey (DLFS) 2000-2013, which, by use of the participants’ personal identification numbers, will be linked to data from a series of Danish national registers. The following registers will be used: Central Person Registry [33], Employment Classification Module [34], Psychiatric Central Research Register [35], and National Prescription Registry [36]. The Danish Labor Force Survey has been conducted all year long since 1994. Each quarter of a calendar year, a random sample of people 15-74 years old is drawn from the Population Statistics Register. An extra sample of unemployed people is drawn from the register-based unemployment statistics (RAM). The samples are divided into 13 equal portions, one for each week of the given quarter, and the persons are invited to be interviewed about circumstances that relate to the reference weeks in question. The participants are also invited to participate in interviews three more times during a period of approximately 15 months after the first interview. Each sample is drawn independently of previous samples, which means that the same person may be sampled in several different quarters. In 2007, the quarterly sample sizes were increased from approximately 20,000 to 40,532 persons. The interviews were conducted by telephone during the time period of the present study and covered various aspects of, inter alia, labor market attachment and working time arrangements [37]. The response rate has decreased with time, from 70% in 2002 to 53% in 2013. The Central Person Registry contains information on gender, addresses, and dates of birth, death, and migrations for every person who is or has been an inhabitant of Denmark sometime between 1968 and the present time. Since 1995, the Psychiatric Central Research Register has covered inpatient, outpatient, and emergency ward visits of all psychiatric hospital departments in Denmark, while the National Prescription Registry covers all redeemed prescriptions at pharmacies in Denmark. A person’s SES, occupation, and industry have been registered annually in the Employment Classification Module since 1975. Persons are classified on the basis of their main income source during a calendar year.

### Exposure Variables

The exposure variables of the present project will be based on responses to the DLFS. The variables will be defined in the same way as in a previous DLFS-based study that examined the association between working time arrangements and ischemic heart disease [38]. The exposure data and exposure variables are described in the previous study [38], as follows: “The labor force surveys gather person-based information on weekly working hours, calculated by adding the hours worked in secondary jobs to the ones worked in a primary job. The participants are asked first how many hours they usually work and then how many hours they worked during the reference week (a predetermined work week, which occurred 1-4 weeks prior to the interview). They are also asked whether and to what extent they work at night. The questions used to gather this information have changed slightly with time. Before 2001, there was no mention of whether meal breaks should be counted as working hours. During 2001-2006, all participants were instructed to exclude meal breaks when they counted their work hours. As of 2007, the time used for meal breaks is to be counted if the person was paid while eating and is to be excluded otherwise. Another peculiarity that was introduced in 2007 is that the participants are asked whether the weekly working hours vary a lot or there are other reasons that make it difficult to provide a meaningful estimate of usual weekly working hours. If they answer ‘yes’ to any of these questions, then ‘average working hours’ is to be used as a proxy for ‘usual working hours’.”

Before 2001, the participants were simply asked whether they worked at night, but from 2001 onward, the question has been whether they worked at night during the last 4 weeks. Until 2006, the response categories were “yes, regularly,“ “yes, occasionally,” and “no, never.“ From 2007 onward, the response categories were expanded to “yes, regularly” (ie, more than half of the working days in the last 4 weeks), “yes, occasionally“ (ie, at least once within the last 4 weeks, but less than half of the working days), and “no, not within the last 4 weeks.”

We will disregard the changes in the data collection routines in the primary analyses of this project. We will define the exposure variables as follows.

#### Weekly Working Hours

In keeping with Kleppa et al [39] and Hannerz and Albertsen [28], we will treat working hours as a categorical variable, with 32-40 hours representing normal weekly working hours, 41-48 hours representing overtime work that lies within the limits of the European working time directive, and 49-100 hours representing overtime work beyond the threshold of the directive. We will base the categorization on the person’s usual working hours.

#### Nighttime Work

Participants who responded either “yes, regularly“ or “yes, occasionally” to the question about nighttime work will be defined as being exposed, and those who responded with “no...“ will be defined as being unexposed to nighttime work.

### Follow-up and Inclusion Criteria

The study will include people who responded to DLFS sometime during the calendar years 2000-2013. The participants will be followed from the end of the calendar year of their baseline interview. The follow-up will end after 5 years or at the time the participant reaches the clinical endpoint of the analysis, emigrates, or dies, or the study period ends (December 31, 2014 for psychotropic medicine; December 31, 2018 for psychiatric hospital treatment), whichever comes first. To be eligible for inclusion, participants should be between 20 and 59 years old at the start of the follow-up period and employed with ≥32 weekly working hours at the time of the interview. People who received psychiatric hospital treatment or redeemed a prescription for psychotropic drugs during the calendar year preceding the start of the follow-up period will be excluded from the analyses. We will moreover exclude all participants who were registered in the Employment Classification Module as unemployed or otherwise not economically active during the main part of the calendar year preceding the start of the follow-up.

### Statistical Analysis

#### Incidence Rates of Redeemed Prescriptions for Psychotropic Medicine and Psychiatric Hospital Treatment

We will use Poisson regression to separately analyze incidence rates of redeemed prescriptions for psychotropic medicine and incidence rates of psychiatric hospital treatment, due to mood disorders, anxiety disorders, or stress-related disorders as a function of weekly working hours (32-40 hours/week, 41-48 hours/week, >48 hours/week), night shift work (Yes vs No), sex, age (10-year categories), calendar time of the interview (2000-2004, 2005-2009, 2010-2013), and SES (legislators, senior officials, and managers; professionals; technicians and associate professionals; workers in occupations that require skills at a basic level; workers in elementary occupations; and gainfully occupied people with an unknown occupation). SES is based on job category, according to the Employment Classification Module, during the calendar year of the baseline interview. The logarithm of person years at risk will be used as offset. Likelihood ratio tests will be used to test the effects that are listed in the section entitled “Hypothesis Tests”. Each of the effects on incidence rates of redeemed prescriptions for psychotropic medicine will be tested at the significance level .01. The effects on incidence rates of psychiatric hospital treatment will be tested at the significance level .05.

**Figure 1:**
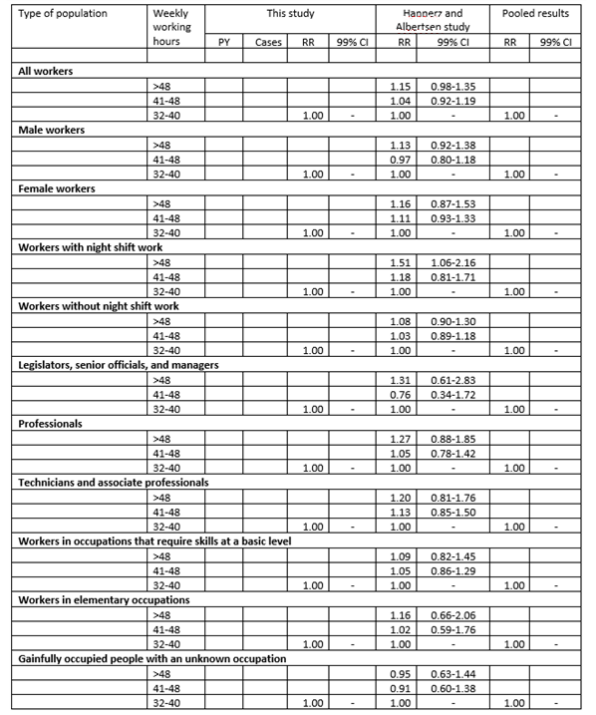
Dummy table: rate ratios (RRs) with 99% CIs for incident use of psychotropic drugs as a function of weekly working hours among employees in Denmark, with reference to the study by Hannerz and Albertsen [23]. PY: person years at risk.

**Figure 2:**
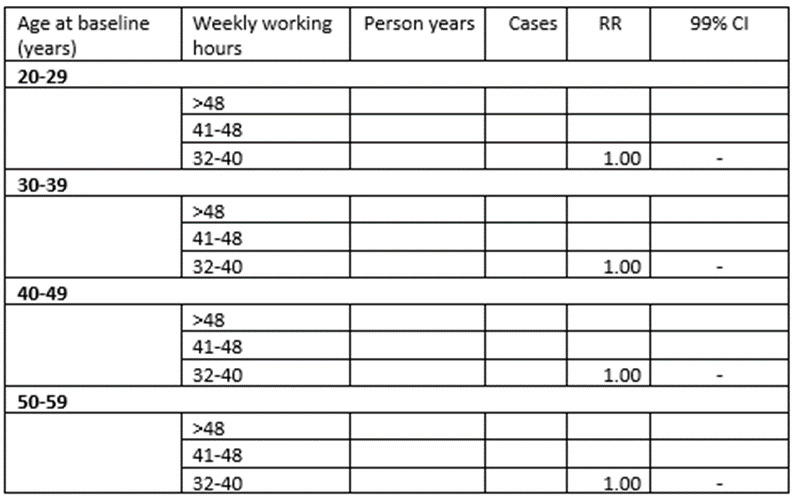
Dummy table: age group–specific rate ratios (RRs) with 99% CIs for incident use of psychotropic drugs as a function of weekly working hours among employees in Denmark in the calendar years 2000-2013.

RRs for redeemed prescriptions of psychotropic drugs as a function of weekly working hours will, thereafter, be estimated by sex, age, night shift work, and SES, and the results will be presented as shown in Figures 1 and 2. As shown in the table, we intend to pool the results of the present study with results from our previous study [23]. The pooled results will be obtained through inverse-variance weighting. The pooled results will provide estimates that are based on the present as well as our previous project. These estimates will afford a higher confidence with regard to the concerned RRs than the present study alone. The statistical significance tests of the present study will, however, only be based on the results of the present project. Since the present study has the same target population as our previous study and the study periods are overlapping, it is likely that some of the participants in our previous study also have participated in the DLFS. Based on the number of participants in our previous study in relation to the number of people in the target population, we expect that approximately 1% of the participants of the present study also participated in our previous study. This overlap will be taken into account in the pooling of the results by use of the following strategy: Before the results are pooled, the standard error of the present study will be multiplied by the square root of (1/(1-x)), where x = 0.01 is the proportion of the participants in the present study that are likely to have participated in our previous study.

**Figure 3:**
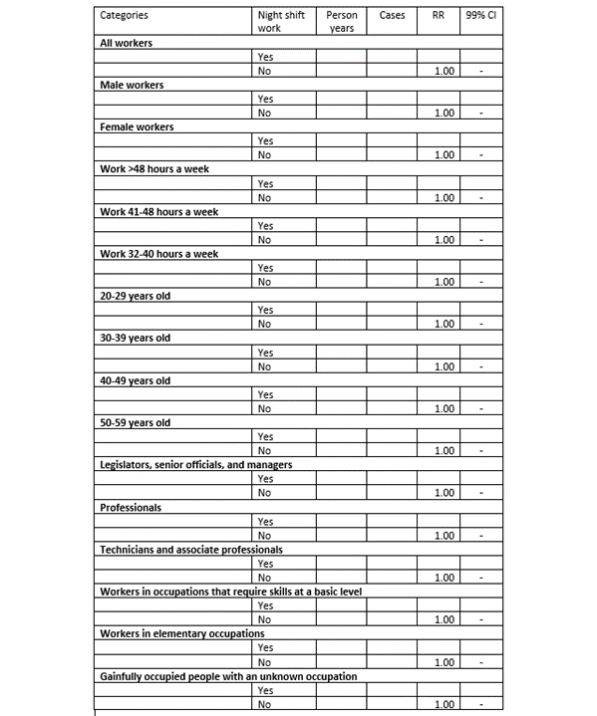
Dummy table: rate ratios (RRs) with 99% CIs for incident use of psychotropic drugs as a function of night work among employees in Denmark 2000-2013.

RRs for redeemed prescriptions of psychotropic drugs as a function of night work will be estimated by sex, weekly working hours, age, and SES, with results presented as shown in Figure 3.

#### Odds Ratio for Antidepressants vs Anxiolytics

This analysis will include all participants who, during the follow-up for psychotropic drugs, redeemed a prescription for either antidepressants or anxiolytics. Logistic regression analysis will be used to estimate the odds that their first redeemed prescription was for antidepressants rather than anxiolytics as a function of night shift work (Yes vs No). The analysis will be controlled for weekly working hours, sex, age, calendar time, and SES. The control variables will be defined as described earlier in the manuscript. A likelihood ratio test will be used to test for a main effect of night shift work. The significance level is set at .05. The estimated odds ratio will be presented with the 95% CI.

#### Odds Ratio for Mood Disorders vs Anxiety Disorders and Stress-Related Disorders

This analysis will include all participants who, during the follow-up, underwent psychiatric hospital treatment for a mood disorder, anxiety disorder, or stress-related disorder. Logistic regression analysis will be used to estimate the odds that their first psychiatric hospital contact during the follow-up was for a mood disorder vs anxiety disorders and stress-related disorders, as a function of night shift work (Yes vs No). The analysis will be controlled for weekly working hours, sex, age, calendar time, and SES. The control variables will be defined as described earlier in the manuscript. A likelihood ratio test will be used to test for a main effect of night shift work. The significance level is set at .05. The estimated odds ratio will be presented with the 95% CI.

### Power Calculations

Based on the National Prescription Registry and Psychiatric Central Research Register, we expect to find approximately 29 new cases of psychotropic drug use and 3.4 new cases of psychiatric hospital treatment per 1000 person years at risk. Based on the number of participants in the DLFS included in previous research [40] and these rates, we expect the follow-up for redeemed prescriptions of psychotropic drugs to encompass 600,000 person years at risk and 17,400 cases, and we expect the follow-up for psychiatric hospital treatments to encompass 700,000 person years at risk and 2400 cases. We expect that 84% of the included participants are working 32-40 hours a week, that 10% are working 41-48 hours a week, and that the remaining 6% are working more than 48 hours a week [41]. We expect that 12.6% of the participants will be categorized as exposed to night shift work [42].

#### Power to Detect Main Effects

**Figure 4:**
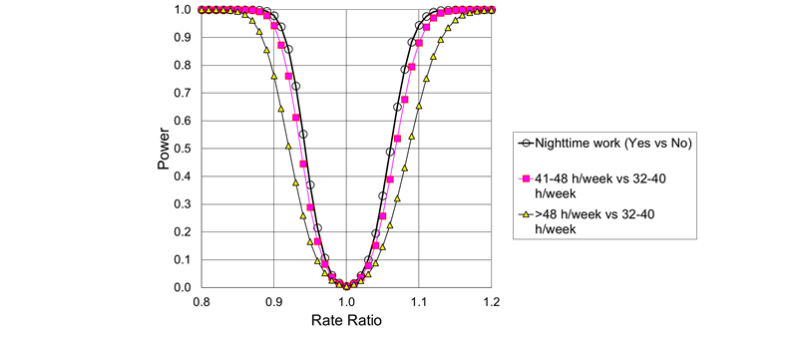
Power to detect main effects of night shifts and long working hours on the rates of new cases of psychotropic drug use, as a function of underlying rate ratios (α=.01).

**Figure 5:**
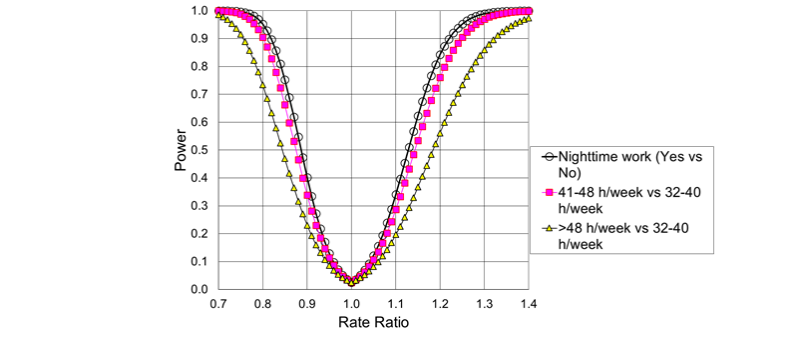
Power to detect main effects of night shift work and long working hours on the rates of new cases of psychiatric hospital treatment for mood disorders, anxiety disorders, or stress-related disorders, as a function of underlying rate ratios (α=.05).

The statistical power for the main effects of night shift work and weekly working hours on the rates of new cases of psychotropic drug use and psychiatric hospital treatment for mood disorders, anxiety disorders, or stress-related disorders, as a function of the underlying RR, is given in Figures 4 and 5. The calculations are based on the expected number of cases, the Poisson distribution, Gauss’ propagation of error formulas, and the central limit theorem.

#### Power to Detect Interaction Effects

In the present project, we calculated the statistical power to detect interaction effects in relation to Cohen w, defined as

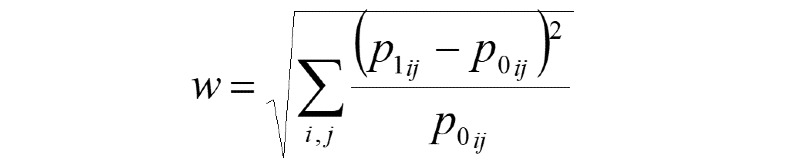



where p_0ij_ and p_1ij_ are the expected proportions of cases that fall into exposure category I, j under the null hypothesis and alternative hypothesis, respectively. According to Cohen, w=0.1 is a small effect, w=0.3 is a medium effect, and w=0.5 is a large effect [43].

The estimated statistical power to detect a small interaction effect (w=0.1) was greater than 99% for each of the interaction tests listed in the section entitled “Hypothesis Tests.” The power calculations were based on the total number of expected cases, the non-central chi-square distribution, and a two-tailed significance level of .01.

These analyses indicate that the power to detect effects of the concerned working time arrangements is sufficient.

#### Power for the Analysis of the Odds for Antidepressants vs Anxiolytics

**Figure 6:**
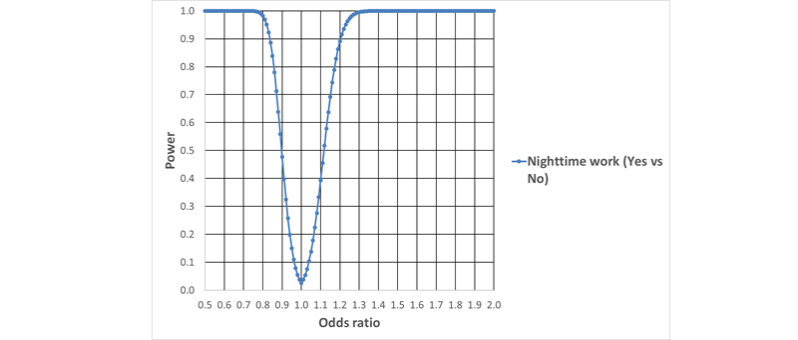
Power to detect an effect of night shift work on the odds of redeemed prescriptions for antidepressants vs anxiolytics, as a function of underlying odds ratios (α=.05).

We expect to find 7900 cases of redeemed prescriptions for antidepressants and 4600 cases of redeemed prescriptions for anxiolytics. The power to detect an effect of night shift work on the odds for antidepressants vs anxiolytics is given in Figure 6. The calculations are based on the expected number of cases, the binomial distribution, Gauss’ propagation of error formulas, and the central limit theorem.

#### Power for the Analysis of the Odds for Mood Disorders vs Anxiety Disorders and Stress-Related Disorders

**Figure 7:**
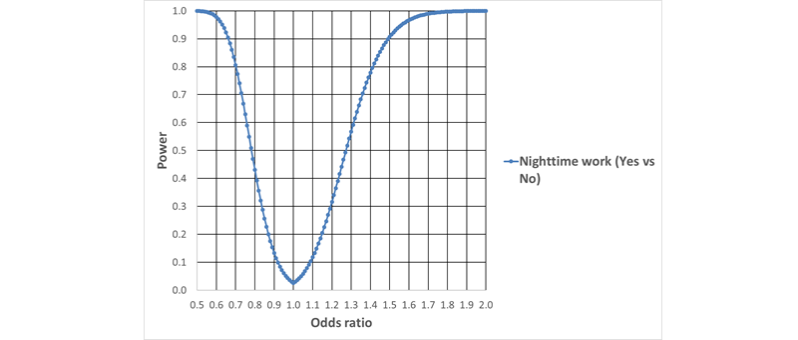
Power to detect an effect of night shift work on the odds of psychiatric hospital treatment for mood disorders vs anxiety disorders and stress-related disorders, as a function of underlying odds ratios (α= .05).

We expect to find 1090 cases of psychiatric hospital treatment for mood disorders and 1310 cases of psychiatric hospital treatment for anxiety disorders and stress-related disorders. The power to detect an effect of night shift work on the odds for mood disorders vs anxiety disorders and stress-related disorders is given in Figure 7. The calculations are based on the expected number of cases, the binomial distribution, Gauss’ propagation of error formulas, and the central limit theorem.

### Sensitivity Analyses

We will conduct a series of sensitivity analyses that will include all of the covariates listed in the statistical analysis section, and all the analyses will have redeemed prescriptions for psychotropic drugs as the endpoint. Only main effects will be considered. The sensitivity analyses will not be tested for statistical significance. Their results may, however, strengthen, weaken, or invalidate statistical conclusions of the primary analyses.

#### Sensitivity Analysis 1: Stable Exposure to Night Shift Work

To determine if the estimated strength of the association between night shift work and redeemed prescriptions for psychotropic drugs increases when the supposedly harmful exposure to night shift work is more stable over time (dose-response association), we will conduct a sensitivity analysis that will only include people who (1) participated in more than one interview, (2) were between 20 and 59 years old during their last interview, (3) were employed 32 or more working hours a week according to their first as well as their last interview, and (4) belonged to the same category in relation to night shift work (yes vs no) during their last interview as they did during their first interview. The follow-up of the included participants will commence at the very end of the calendar year of their last interview. The statistical model will otherwise be the same as in the primary analysis.

#### Sensitivity Analysis 2: Stable Exposure to Weekly Working Hours

Similarly, to determine if the estimated strength of the association between working hours and redeemed prescriptions for psychotropic drugs increases when exposure is more stable over time, we will conduct a sensitivity analysis that will only include people who (1) participated in more than one interview, (2) were between 20 and 59 years old during their last interview, (3) were employed 32 or more working hours a week according to their first as well as their last interview, and (4) did not move more than one step among the ordered working time categories between the first and last interview. The included participants will then be categorized by weekly working hours into 32-40 hours/week, 41-48 hours/week, and ≥49 hours/week, according to the mean of the reported usual working hours during their first and last interview. The follow-up of the included participants will commence at the very end of the calendar year of their last interview. The statistical model will otherwise be the same as in the primary analysis.

#### Sensitivity Analysis 3: Occasional vs Regular Night Shift Work

We want to know if the estimated strength of association between night shift work and redeemed prescriptions for psychotropic drugs is greater among participants with regular night shift work than it is among participants with occasional night shift work. We will therefore conduct a sensitivity analysis where we divide night shift work into three categories (no; yes, occasionally; yes, regularly) and then estimate the RRs for the contrasts of ”yes, occasionally“ vs “no” and ”yes, regularly“ vs ”no“. The statistical model and inclusion criteria will otherwise be the same as in the primary analysis.

#### Sensitivity Analysis 4: Inclusion of Workers with 28-31 Working Hours a Week

In the primary analysis, we will only include employees who usually worked ≥32 hours a week. There are, however, relatively large groups of night shift workers in nursing homes and home care whose standard full-time work schedules (eg, 7 night shifts, 7 days off-duty) imply an average of only 28 working hours a week. We want to know if the estimated effect of night shift work on the rates of new cases of psychotropic drug use would change if our reference group was changed from 32-40 hours/week to 28-40 hours/week. We will therefore conduct a sensitivity analysis with a redefined inclusion criterion at ≥28 hours/week and a redefined reference group at 28-40 hours/week. The statistical model will otherwise be the same as in the primary analysis.

#### Sensitivity Analysis 5: Controlling for Possible Bias due to Preexisting Mental Health Problems

**Figure 8:**
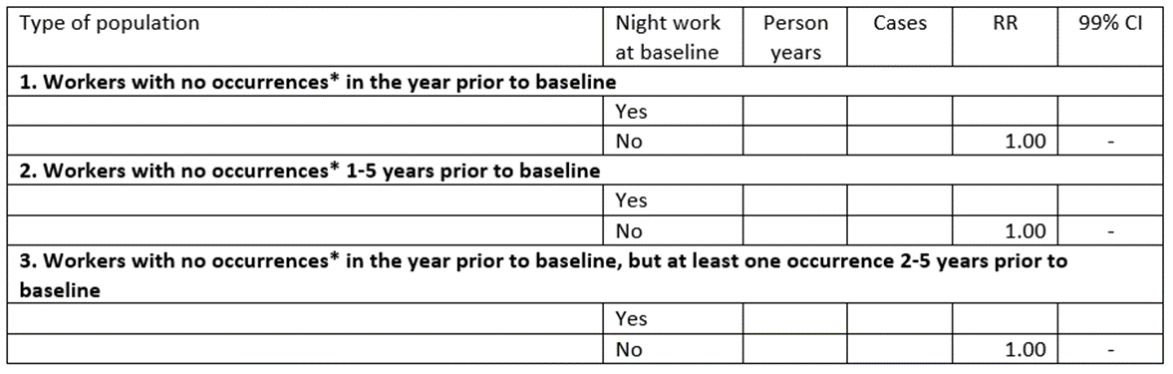
Dummy table: rate ratios (RRs) with 99% CIs for incident or recurrent use of psychotropic drugs, as a function of night work among employees in Denmark 2004-2013. Populations 2 and 3 are disjointed and exhaustive subsets of population 1. *The term occurrences refers to “occurrences of redeemed prescriptions for psychotropic medicine or psychiatric hospital treatment.”.

**Figure 9:**
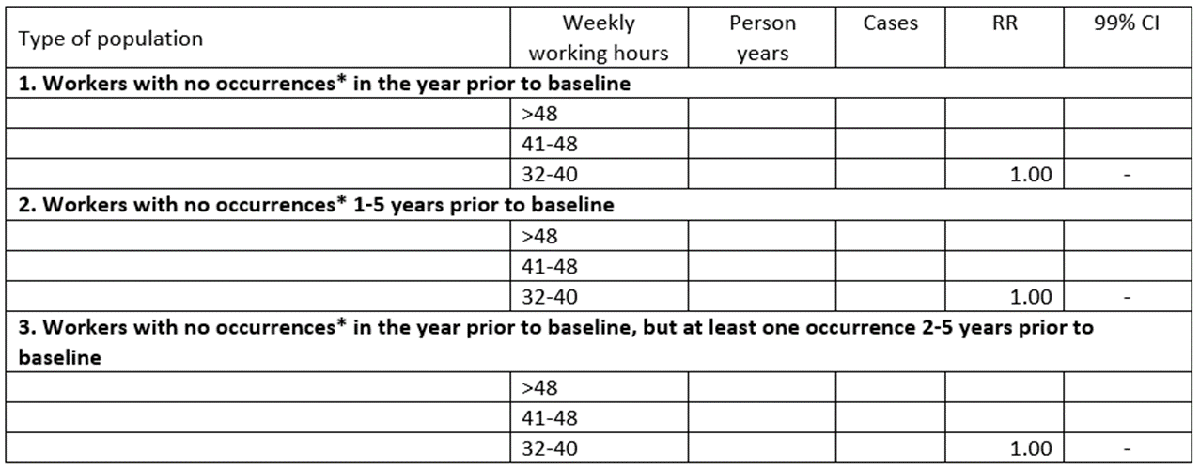
Dummy table: rate ratios (RRs) with 99% CIs for incident or recurrent use of psychotropic drugs, as a function of weekly working hours among employees in Denmark 2004-2013. Populations 2 and 3 are disjointed and exhaustive subsets of population 1. *The term occurrences refers to “occurrences of redeemed prescriptions for psychotropic medicine or psychiatric hospital treatment.”.

In the primary analysis, we will exclude participants who received psychiatric hospital treatment or redeemed a prescription for psychotropic drugs during the calendar year preceding the start of the follow-up period. It is, however, possible that the results of the primary analysis will be influenced by cases that occurred earlier than one year prior to baseline. To explore this possibility, we will conduct a sensitivity analysis in which the sample is stratified into two cohorts. The first cohort will exclude all participants who underwent psychiatric hospital treatment or redeemed a prescription for psychotropic drugs some time during the 5-year period prior to the start of follow-up. The second cohort will consist of the participants who were excluded from the first cohort due to psychiatric hospital treatment or redeemed prescription for psychotropic drugs within 2-5 years prior to the start of the follow-up. Participants who underwent psychiatric hospital treatment or redeemed a prescription for psychotropic drugs some time during the 1-year period prior to the start of follow-up will still be excluded. As a genetic or social disposition might increase the development of mental illness in response to an exposure such as night shift work, the second cohort will supplement the primary analyses with information on the effect in people who have previously been treated for mental health problems. This particular analysis will only include participants who lived in Denmark throughout the 5-year period of concern. Moreover, it will only include people who participated in DLFS sometime during the calendar period 2004-2013. The statistical methods and inclusion criteria of the analysis will otherwise be the same as in the primary analysis. The results will be presented as shown in Figures 8 and 9. The results of the first cohort will be interpreted as incidence RRs, while the results of the second cohort will be interpreted as relapse RRs.

#### Sensitivity Analysis 6: Controlling for the Industrial Sector

In order to pool results of the present study with results obtained in our previous study (see Figure 1) we will use the same covariates in the primary analysis of the present study as we did in our previous study [23]. The primary analysis therefore controls for an occupational-based SES, but it does not control for the industrial sector, which has been shown to be a predictor for mood disorders in the general working population of Denmark [44]. We want to know if the results of the present study will change if we add the industrial sector to the model and will therefore conduct a sensitivity analysis where we first control for and thereafter stratify by industrial sector. The statistical methods and inclusion criteria of the analysis will otherwise be the same as in the primary analysis. The industrial groups will be classified as shown in Table 1. The coding of the industries is based on the industrial classification DB93 [45] in the calendar years 1999-2002, DB03 [46] in 2002-2007, and DB07 [47] in 2008-2013.

**Table 1:** Industrial groups coded according to the subclassifications within the main classifications of DB93, DB03, and DB07.

Industrial group	Classification		
	DB93^a^	DB03^b^	DB07^c^
Agriculture, forestry, hunting, and fishing	A+B	A+B	A
Manufacturing, mining, and quarrying	C+D	C+D	B+C
Construction	F	F	F
Wholesale and retail trade; repair of motor vehicles and motorcycles	G	G	G
Transporting and storage	I	I	H
Accommodation and food service activities	H	H	I
Human health and social work activities	N	N	Q
Other			
Missing	-	-	-

^a^for years 1999-2002.

**^b^**for years 2002-2007.

^c^for years 2008-2013.

#### Sensitivity Analysis 7: Estimated RRs Without Exclusion of Prevalent Cases

In this sensitivity analysis, we will estimate the RRs for redeemed prescriptions for psychotropic drugs as a function of night shift work and weekly working hours without exclusion of prevalent cases. The statistical methods and inclusion criteria of the analysis will otherwise be the same as in the primary analysis.

## Results

We expect the analyses to be completed by the end of 2020 and the results to be published in 2021.

## Discussion

In the present study protocol, we give a complete description of the hypotheses and statistical methods of a project aimed at investigating night shift work and long working hours as predictors for mental ill health in the general population of Denmark. To reduce the risk of hindsight bias and within-study selection bias, the protocol will be peer reviewed and published before we link the exposure data to the outcome data of the project. The statistical analyses are thereby blinded in the sense that all hypotheses, inclusion criteria, significance levels, and statistical models will be completely defined before we look at any relation between working time arrangements and psychotropic drugs or psychiatric hospital treatment in the datasets at hand. It should, however, be noted that the exposure data of the project have previously been analyzed in relation to circulatory disease [38,40,48,49], injuries [50], and all-cause mortality [41,42].

The clinical endpoints of the study as well as the censoring events (deaths and emigrations) will be ascertained through data in national registers, which cover all residents of Denmark. Since the outcome data are based on registers rather than follow-up interviews, we have minimized the risk of bias from missing follow-up data.

Our power calculations indicate that the power of the study is sufficiently large to test overall effects of weekly working hours and night shift work on the overall incidence rates of psychotropic drug usage and psychiatric hospital treatment for mood disorders, anxiety disorders, or stress-related disorders. It is, moreover, sufficiently large to test for interaction effects between working time arrangements on one hand and sex, age, and SES on the other on the incidence rates of psychotropic drug usage. The power to detect a difference in the distribution of mood disorder vs anxiety disorder and stress-related disorder diagnoses between participants with and without night shift work is, however, quite low. Hence, for this particular analysis, the absence of a statistically significant effect cannot be interpreted as the absence of a clinically important effect. It is, however, of interest to see if the estimated odds ratio for mood disorder vs anxiety disorder and stress-related disorder diagnoses points in the same or opposite direction of the odds ratio for antidepressants vs anxiolytics. If it points in the same direction, it will weaken the hypothesis of “different practices for prescription of psychotropic drugs to people working in shifts compared to non-shift workers to avoid side effects that may impede wakefulness during night shifts.”

An advantage of the study is that the questionnaires asked not only for the hours worked in the participants’ primary jobs but also for the hours worked in secondary jobs, which enabled us to categorize the participants’ working hours on the basis of the sum of the hours worked in their primary and secondary jobs. “If we had disregarded hours worked in secondary jobs, then 25.0% of the workers working 49-100 hours a week would have been misclassified as working less than 49 hours and 24.6% of the workers working 41-48 hours a week would have been misclassified as working less than 41 hours” [41].

A drawback of the questionnaires is that they did not ask for the duration of the exposure. We only know the participants’ usual working hours around the time of the interview and whether they had worked at night during a 4-week period preceding the interview. It has, however, been shown that working time arrangements tend to be quite stable over time [28]. Moreover, it has been judged that the exposure to shift work as well as to long working hours are stable enough in the Danish labor force to make 5-year follow-up studies worthwhile, even if the exposure is only measured at a single timepoint [28].

Since this is an observational study, we cannot ignore the possibility of bias due to self-selection into the various working time categories. It is possible that a worker’s decision and ability to work at night or to work long hours depend on his or her working environment, lifestyle, and mental health. This may be especially true when it comes to the decision and ability to work long hours. It is possible that employees with poor mental health tend to be more reluctant to work long hours than employees with good mental health, which would bias the results towards decreased rates of mental ill health among employees with long working hours. It is, however, also possible that employees with poor mental health tend to be less reluctant to work long hours. It has, for example, been shown that workaholics are highly overrepresented among people with long working hours [51]. It has, moreover, been shown that workaholism is associated with obsessive-compulsive disorders, attention deficit hyperactivity disorder, anxiety, and depression [52]. To mitigate potential healthy worker effects, we will exclude employees who redeemed a prescription for psychiatric drugs in the year preceding baseline. A residual healthy worker effect is possible since mental health problems may exist also among employees who do not use prescription drugs. In our previous studies, sensitivity analyses, in which participants with poor self-rated health at baseline were excluded, suggested that any such bias is small [23,28].

It should also be noted that participation in the Danish labor force surveys is voluntary, which provides an additional potential for self-selection bias (non-response bias). We know that response rates to questionnaires on work environmental issues and health in Denmark depend on calendar year [28], age, sex, marital status, SES, and ethnic background [53-55]. It is possible that the response rates also depend on the persons’ working time arrangements as well as their mental health. If the response rates among people with poor mental health depend on their working time arrangements, the results of our analysis will be biased. Any such bias will, however, be mitigated by our decision to exclude prevalent cases, and it will be further mitigated by our decision to control for calendar year of the interview, age, sex, and SES.

Another reason to control for calendar year of the interview, age, sex, and SES is that each of these factors has been associated with indicators of mental ill health [56-60].

It has been suggested that smoking [61,62] and overweight [63] are risk factors for depression. Unfortunately, the labor force surveys do not contain any data on BMI or smoking. We can therefore not control for these factors in the analyses. We have, however, previously examined the relationship between night shift work, long working hours, and prevalences of smoking, overweight, and BMI in our target population [38]. The study indicated that weekly working hours are independent of smoking and BMI. However, among employees with vs without night shift work, we found that the prevalences were higher among night shift workers for smoking (25.8% vs 21.6%), overweight (38.4% vs 34.5%), and obesity (15.4% vs 12.7%). The risk ratio of depression has been estimated at 1.46 (95% CI 1.03-2.07) for smokers vs non-smokers [61], 1.08 (95% CI 1.02-1.14) for overweight vs normal weight, and 1.57 (95% CI 1.23-2.01) for obesity vs normal weight [63]. Based on these numbers and some high school algebra, we estimate that the effect of not controlling for smoking and BMI will bias an estimated RR of depression among employees with vs without night shift work upwards by a factor of 1.03, and this needs to be taken into account when the results of the present study are evaluated.

Another drawback of the labor force surveys is that they do not contain any data on work-environment factors. A sensitivity analysis in a previous study showed, however, that after adjustment for age, sex, and SES, the RR for incident use of psychotropic drugs as a function of working time arrangements did not change when the analyses were further controlled for job satisfaction and job insecurity [23,28].

## Abbreviations

DLFS: Danish Labor Force Survey.
OECD: Organization for Economic Co-operation and Development.
RR: rate ratio.
SES: socioeconomic status.
